# A 10-Year Retrospective Review of Prenatal Applications, Current Challenges and Future Prospects of Three-Dimensional Sonoangiography

**DOI:** 10.3390/diagnostics11081511

**Published:** 2021-08-21

**Authors:** Tuangsit Wataganara, Thanapa Rekhawasin, Nalat Sompagdee, Sommai Viboonchart, Nisarat Phithakwatchara, Katika Nawapun

**Affiliations:** Department of Obstetrics and Gynecology, Faculty of Medicine Siriraj Hospital, Bangkok 10700, Thailand; rthanapa@gmail.com (T.R.); pear_nalat@hotmail.com (N.S.); poopae.alek@hotmail.com (S.V.); nisaratp@gmail.com (N.P.); katika.nawapun@gmail.com (K.N.)

**Keywords:** three-dimensional sonoangiography, three-dimensional power Doppler, three-dimensional ultrasound, flow-volume index, prenatal, fetus, twins, placenta, umbilical cord, cervix

## Abstract

Realistic reconstruction of angioarchitecture within the morphological landmark with three-dimensional sonoangiography (three-dimensional power Doppler; 3D PD) may augment standard prenatal ultrasound and Doppler assessments. This study aimed to (a) present a technical overview, (b) determine additional advantages, (c) identify current challenges, and (d) predict trajectories of 3D PD for prenatal assessments. PubMed and Scopus databases for the last decade were searched. Although 307 publications addressed our objectives, their heterogeneity was too broad for statistical analyses. Important findings are therefore presented in descriptive format and supplemented with the authors’ 3D PD images. Acquisition, analysis, and display techniques need to be personalized to improve the quality of flow-volume data. While 3D PD indices of the first-trimester placenta may improve the prediction of preeclampsia, research is needed to standardize the measurement protocol. In highly experienced hands, the unique 3D PD findings improve the diagnostic accuracy of placenta accreta spectrum. A lack of quality assurance is the central challenge to incorporating 3D PD in prenatal care. Machine learning may broaden clinical translations of prenatal 3D PD. Due to its operator dependency, 3D PD has low reproducibility. Until standardization and quality assurance protocols are established, its use as a stand-alone clinical or research tool cannot be recommended.

## 1. Background

Two- and three-dimensional ultrasounds (2D US and 3D US, respectively) have been well adopted for structural and functional assessments of the fetus, the umbilical cord and placenta, and the cervix during pregnancy [1]. Real-time ultrasound is suitable for point-of-care (POC) management, especially in early pregnancy. Tomographic ultrasound imaging is particularly useful for the 3D US display of the fetal brain. Subsequent volumetric analyses of the acquired 3D US data with semi-automatic Virtual Organ Computed-aided AnaLysis (VOCAL; GE, Kretztecink AG, Zipf, Austria) are standardized [2]. Color Doppler (CD), power Doppler (PD), and high-definition flow (HDF) display flow data superimposed on a B-mode image. However, they are less accurate than angiography because (1) CD and PD exaggerate the size of vessels, and (2) Doppler flow detection is obscured by the grey-scale signal of the overlying tissue [3]. In addition, as the course of a blood vessel is not always within a single examination plane, a 2D Doppler image is not easily reproduced in follow-up comparisons using the same morphological landmarks. Computed tomographic angiography and magnetic resonance angiography can reconstruct the courses of vessels in three orthogonal planes. Nevertheless, computed tomographic angiography is invasive, and its ionizing radiation can damage a developing embryo [4]. Moreover, magnetic resonance angiography requires the intravascular injection of gadolinium-based contrast agents, which should be used only when essential [4]. Furthermore, the motion artifacts that are universally found in computerized tomography and magnetic resonance images of smaller vessels limit their use during the first trimester of pregnancy [5,6].

Spectral or pulsed-wave Doppler (PWD) converts frequency shifts between the reflected sound waves and the short-emitted pulses to velocities using the Doppler equation. PWD also displays the waveforms of a spectrum of frequencies. Only the Doppler shifts recorded from an operator-defined small area (sample volume/gate) are recorded and expressed as velocity indices during cardiac systole and diastole, i.e., the pulsatility index, resistance index, and systolic/diastolic ratio. Standardized measurement and quality assurance for obstetric PWD have been established [7]. Tissue Doppler imaging uses PWD principles to measure the velocity/amplitude of myocardial motion. Historically, blood volume within the placenta was estimated via ex vivo injection studies [8]. It is now possible to non-invasively estimate the moving blood volume, regardless of its velocity, within the tissue morphology (flow-volume data) without the need for the intravascular injection of contrast agents. The ultrasound virtual-reality display of the biometry, course, and branching patterns of blood vessels is called 3D sonoangiography [9]. It is also commonly known as 3D power Doppler (3D PD); the initialisms CD, PD, and HDF are also used.

The basic principles of 3D PD are as follows. Color Doppler displays the direction and high-velocity flow of larger vessels in an array of colors. Power Doppler sensitively displays low-velocity flow within smaller vessels in an array of monochromic strengths [10]. By extending the usable dynamic range of the equipment, PD sensitively detects flow over the grey-scale noise with less influence from the angle of insonation and less aliasing [11]. While high-definition flow sensitively displays both direction and velocity in an array of colors, it is more susceptible to aliasing. Novel Doppler modalities have lowered the limit of (flow) detection by enabling signal extraction at a more rapid frame rate. This allows for the production of high-resolution images displaying slow blood flow in the smaller parenchymal veins, venules, and capillaries uniquely found in the placenta and the renal cortex. The current slow-flow modalities, i.e., SlowflowHD (GE GmbH, Vienna, Austria) and Superb Microvascular Imaging (SMI; Canon Medical Systems, Tustin, CA, USA), are not yet compatible with 3D PD [12,13,14].

The archived flow-volume data can be adjusted for intensity, re-rendered, and re-analyzed. Post-scan thick slicing further improves the image quality by eliminating overlapping morphological structures. Elimination of the tissue opacity that obscures the flow image and enhancement of the morphological organ/vascular boundary and cavitation are possible with rendering algorithms that detect abrupt changes in tissue acoustic impedance, such as HDLive Silhouette (GE, Milwaukee, WI, USA) and Hyaline (Mindray, Shenzhen, China) [15,16]. Histogram vascularity indexing (i.e., vascularization index, flow index, and vascularization-flow index) was originally used for the “sonobiopsy” of the endometrium because it provides a broader perspective of parenchymal perfusion characteristics than PWD [17]. The present study aimed to (a) summarize the technical aspects, (b) determine the additional advantages, (c) identify the current challenges, and (d) predict the future trajectories of 3D PD for prenatal assessments.

## 2. Methods

A literature search was conducted of the PubMed and Scopus databases for the past decade (July 2011 to June 2021). This is the period when the onset of the technological commercialization and near-maturation of 3D PD made it a viable tool for research and clinical-care applications. The search terms were three-dimensional sonoangiography, three-dimensional power Doppler, three-dimensional ultrasound, flow-volume index, prenatal, fetus, twins, placenta, umbilical cord, and cervix. The inclusion criteria were published studies on prenatal assessment, while the exclusion criteria were non-English publications.

## 3. Results

In all, there were 538 articles that met one or more of the search terms. After manual review, 307 were found to address our objectives. Those studies had a broad range of study methodologies, indications, and technical perspectives of prenatal 3D PD. The ones with qualitative (descriptive) methodologies (the case reports and case series) described clinical feasibility. The technology seemed to be most promising for POC diagnoses of the placenta accreta spectrum. The papers that employed quantitative methodologies (the cross-sectional and case–control studies) primarily focused on the global 3D PD vascularity indices of the first-trimester placenta for early prediction of preeclampsia, and the focal 3D PD vascularity indices of the second- and third-trimester placenta to guide prenatal management of intrauterine growth restriction. Unfortunately, the heterogeneity of the 307 publications was too broad to conduct meaningful statistical analyses. Therefore, only publications with important findings are presented herein, using a descriptive format. The authors’ original fetal and extra-fetal 3D PD images are also used to augment the narration.

### 3.1. Fetal Central Nervous System

**Figure 1:**
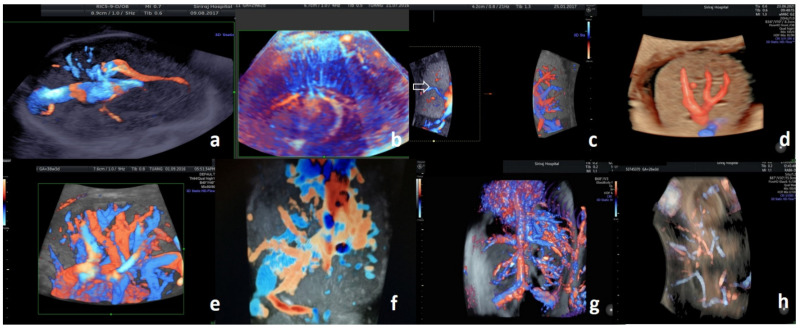
Fetal assessment with three-dimensional sonoangiography. (**a**) Vein of Galen aneurysmal malformation of a fetus at 30 weeks of gestation; thick-slice, three-dimensional, high-definition flow (3D HDF). (**b**) Deep medullary veins of a fetus at 29 weeks of gestation; thick-slice 3D HDF. (**c**) Diminished vasculatures (arrow) of right lung with primary dysplasia, compared with the left normal lung, in left and right lung of a fetus at 29 weeks of gestation; thick-slice 3D HDF. (**d**) Primitive hepatic vasculature of a fetus at 22 weeks of gestation; HDLive Silhouette (GE, Milwaukee, WI, USA), thick-slice 3D HDF. (**e**) Mature hepatic vasculatures of a fetus at 38 weeks of gestation; thick-slice 3D HDF. (**f**) Confluent vasculatures of hepatic hemangioma of a fetus at 30 weeks of gestation; thick-slice 3D HDF. (**g**) Complex visceral vasculatures of a fetus at 20 weeks of gestation; thick-slice 3D HDF. (**h**) Primitive visceral vasculatures of an acardia at 26 weeks of gestation; HDLive Silhouette (GE, Milwaukee, WI, USA), thick-slice, 3D HDF.

Early ultrasound recognition of congenital anomalies and acquired in-utero brain damage through the sagittal cranial suture window may permit timely intervention and obviate long-term neurodevelopmental disability [18,19]. The 3D US parameters for fetal brains have been standardized [20]. An abnormal biometry or a defective course of intracranial vessels can be realistically appreciated with 3D PD. Dilatation of the straight sinus (Figure 1a), especially if accompanied by ventriculomegaly or other major brain abnormalities, is suggestive for vein of Galen aneurysmal malformation [21]. A defective pericallosal artery, visualized by Doppler mapping from 11 weeks of gestation, is suggestive for maldevelopment of the corpus callosum, which is the largest white matter structure in the brain [22]. Most of the other white matter abnormalities are subtle and challenging for prenatal ultrasound diagnosis.

The deep medullary veins of white matter can be prenatally visualized as fine parenchymal vasculatures draining from pia mater to subependymal veins. There is a parallel distribution pattern near the body and inferior horn (Figure 1b), and a radial distribution pattern near the frontal horn or trigone of the lateral ventricle [23,24]. Disruption of deep medullary vein vasculatures is associated with congenital venous malformations, stroke (from physical trauma, engorgement, or thrombosis), viral infections, neoplasms, and metabolic disorders of the fetus [25]. Clinical interpretation of isolated deep medullary vein abnormalities is not possible because the functional assessment of neuronal connectivity is currently not available via US [26].

### 3.2. Fetal Intrathoracic and Intra-Abdominal Viscera

Fetal pulmonary vasculatures are realistically appreciated with 3D PD (Figure 1c). The administration of betamethasone or dexamethasone in women at risk of preterm birth between 24 and 33 weeks of gestation accelerates fetal lung maturation. The response can be demonstrated with 2D CD semi-quantitation of pulmonary parenchymal perfusion [27,28]. Defective 2D PD pulmonary vasculatures of third-trimester fetuses affected by congenital diaphragmatic hernia (CDH) can predict lung hypoplasia at the time of birth [29]. Pulmonary perfusion can be realistically visualized by 3D HDF after 13 weeks of gestation [30]. Moderate and severe CDH significantly affect the pulmonary volume and arterial pressure of the fetus, and thus increase the risk of ventilation/perfusion mismatch at the time of birth. Prenatal alleviation of these CDH-related changes after a fetal intraluminal tracheal occlusion procedure can be demonstrated with sequential measurements of pulmonary flow-volume indices [31,32,33,34].

The fetal liver receives its oxygenated blood from the placenta. Fifty percent of the umbilical venous blood bypasses to the right atrium via the ductus venosus and inferior vena cava, and the PWD velocity indices represent cardiac preload [35]. The rest of the umbilical venous flow is distributed in a tree-like pattern with an orderly segmentation grid to both liver lobes. Therefore, any parenchymal disruption of 3D PD is readily discernable (Figure 1d–f). In cases of omphalopagus twinning, the sharing of large hepatic vessels that are prenatally visualized with 3D PD may be predictive for hemorrhagic morbidity during surgical separation [36,37].

### 3.3. Fetal Mediastinal and Retroperitoneal Great Vessels

Cardiac examination planes derived from spatial temporal image correlation have been standardized in accordance with 2D CD prenatal diagnostic criteria [38]. The ascending aorta, pulmonary trunk, pulmonary veins, superior vena cava, and inferior vena cava are great vessels with direct associations with the heart. They can be realistically displayed with 3D PD [39,40,41]. Three-dimensional printing of these vessels aids in understanding the development of complex anomalies of the cardiac inflows and outflows (Figure 1g,h) [42,43,44].

### 3.4. Fetal Tumors

Tumors with prenatal onset are rare. Cardiac burden, high-output failure, and permanent myocardial dysfunction are more common in tumors with higher vascularity, namely, sacrococcygeal teratomas [45,46]. Integration of a sacrococcygeal teratoma’s 3D PD vascularization index with the tumor-to-fetal volume ratio, tumor growth rate, and combined cardiac output indexed to the estimated fetal weight may aid in prognostication, a decision to intervene prenatally, and the timing of the delivery [47].

Overall, most cases with a prenatally diagnosed congenital pulmonary airway malformation (CPAM) carry a favorable prognosis, with over 95% neonatal survival and up to 50% spontaneous antenatal resolution [48]. However, CPAM with hydrops has over 95% perinatal death. In-utero thoracoamniotic shunting and open fetal surgery with lobectomy may improve survival for the macrocystic and microcystic types, respectively [48]. The CPAM volume (measured with 3D US) to fetal head circumference ratios have been linked with prognosis [49]. The 3D US volume to head circumference ratios of prenatally detected pulmonary sequestration are also predictive of perinatal outcomes [50]. However, our search did not find any publication on the use of 3D PD for prognosticative purposes for cases of prenatally detected CPAM or pulmonary sequestration. Primary cardiac tumors in the fetus are extremely rare [51]. The most common congenital cardiac tumor is rhabdomyoma, which has a strong genetic predilection. A positive genetic diagnosis can assist in counseling and planning for neonatal treatment [52]. Our review did not find any publication relating to the prenatal evaluation of cardiac tumors with 3D PD.

### 3.5. Placenta

**Figure 2:**
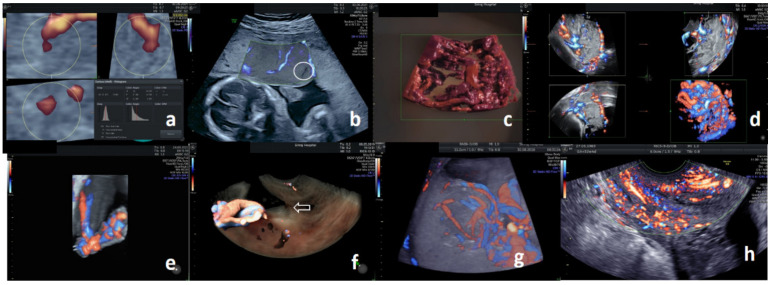
Extra-fetal assessment with three-dimensional sonoangiography. (**a**) Histogram flow-volume indices of the placenta at 26 weeks of gestation; three-dimensional (3D) power Doppler (PD) with a spherical representation of the entire placental vascular tree. (**b**) Normal parenchymal vasculatures of the placenta at 24 weeks of gestation; SlowflowHD (GE GmbH, Vienna, Austria). Note the virtual absence of flow in the lake (circle). (**c**) Confluent parenchymal vasculatures of placenta percreta at 29 weeks of gestation; HDLive Silhouette (GE, Milwaukee, WI, USA), thick-slice, monochrome 3D high-definition flow (HDF). (**d**) Complicated vasculatures involving the entire thickness of the placenta, with extension to the myometrial–bladder interface of placenta percreta, at 29 weeks of gestation; orthogonal multiplanar 3D HDF. (**e**) Velamentous umbilical cord insertion at 25 weeks of gestation; thick-slice 3D HDF. Note the transition from coiled umbilical vessels to chorionic vessels with the artery (blue) crossing over the vein (red). (**f**) Marginal placenta previa at 28 weeks of gestation; HDLive Silhouette (GE, Milwaukee, WI, USA), thick-slice 3D HDF. Note the proximity of the velamentous umbilical cord insertion to the internal cervical os (arrow). (**g**) Feeding vessels of chorioangioma at 29 weeks of gestation; thick-slice 3D HDF. (**h**) Parenchymal vasculatures of a normal cervix at 32 weeks of gestation; two-dimensional HDF.

Large and highly vascularized placenta may be more adaptive to maternal–fetal interaction and more favorable to pregnancy [53]. The entire first-trimester placenta can be covered with a single acquisition, and a lower placental volume was linked with preeclampsia and intrauterine growth restriction [54,55,56]. Lower first-trimester placental vascularity indices yield the highest accuracy for the prediction of early-onset preeclampsia when integrated with other parameters [57,58,59,60]. Lower placental 3D PD indices of the volume arbitrarily sampled underneath the placental insertion of the umbilical cord of the second-trimester placenta (Figure 2a) are more predictive for intrauterine growth restriction [61,62]. A lack of consensus on standardization and quality reassurance, especially for sonobiopsies that can represent the entire placenta, precludes the clinical translation of placental vascularity indices [63,64].

Sonolucent placental lakes have the lowest positive predictive value (PPV) for placenta accreta spectrum (PAS), especially when there are no 2D PD signals with a sufficiently low threshold (Figure 2b) [65]. The presence of turbulent/aliasing 2D CD signals suggests PAS; however, a focal area of complicated vasculatures may be obscured by the vast area of the vascularized third-trimester placenta [66]. The 3D PD uniquely acquires deeper examination planes and better depicts numerous coherent vessels affecting the entire placental thickness with extension to the uterine serosa–bladder interface (Figure 2c,d), which is the best single PAS diagnostic criterion (97% sensitivity, 92% specificity, and 77% PPV) [67,68]. The majority of cases of PAS also show multiple characteristic features on ultrasound. Integration of 3D PD findings into a scoring system further improved the diagnostic PPV and inter-rater agreement to nearly 90%, which is comparable with that achievable with a magnetic resonance imaging diagnosis [69,70,71,72,73].

Abnormal proliferation of trophoblastic cells derived from the placenta can cause gestational trophoblastic neoplasia. The neoplasm is highly vascular and is associated with massive hemorrhage. Selective arterial embolization can effectively control the bleeding, and 3D PD using glass body surface rendering may aid in the POC color flow mapping of a uterine arteriovenous malformation with high vascularity associated with a gestational trophoblastic neoplasia [74].

### 3.6. Umbilical Cord

Reconstruction with 3D PD may aid in prenatal assessment of the umbilical cord (Figure 2e,f) [37]. Simultaneous orthogonal US display may shorten the time and risks associated with in-utero procedures [75,76,77]. Virtual placentoscopy using 3D PD can demonstrate residual chorionic vessels following fetoscopic photocoagulation for severe twin-to-twin transfusion syndrome and chorioangioma (Figure 2g) [78,79,80].

### 3.7. Cervix

A short cervix in high-risk women predicts a spontaneous preterm birth before 34 weeks of gestation that could be prevented with progesterone administration. The clinical decision for an asymptomatic short cervix in low-risk women may be aided by the US elastography index and strain pattern score of the cervix [81]. The entire cervix can be covered with a single acquisition (Figure 2h); a smaller volume and a higher flow-volume index independently elevate the risk for a spontaneous preterm birth [82,83,84]. Algorithmic integration of elastography and flow-volume data with magnetic resonance elastography reduces false positive results [85]. Elastographic sonoangiography has recently become available for research purposes, and its clinical validation is underway.

### 3.8. Technical Perspectives

The quality of ultrasound assessments relies on techniques (of the operators) and physics (of the equipment). Choosing the right ultrasound transducer is vital. Tissue penetration is primarily determined by the center frequency of the transducer: the higher the frequency, the shallower the penetration. A mechanical, automated sweeper is increasingly being preferred, with free-hand and 2D array techniques for a more flexible volume acquisition in challenging cases. The acquisition (sweeping) speed primarily affects the quality of the volume data, whereas the angle of insonation and Doppler settings primarily affect the quality of the flow data. The most influential Doppler settings are gain, signal power, and pulse repetition frequency (PRF), in decreasing order of influence. The PRF is the Doppler sampling frequency (kilohertz) emitted from the transducer, which determines the maximum Doppler shifts obtainable. Therefore, the equipment should be individually optimized to reduce artifacts for an accurate real-time diagnosis. It should begin with a Doppler gain and signal power to produce no Doppler signals at the lowest settings, but the highest recordable indices at the maximum settings. Then, the PRF is adjusted until the Doppler signal is free of aliasing and is measurable at all of the different settings. Wall motion filtering removes Doppler signals with outlier frequencies and subtly improves the image quality without having a significant impact on the 3D PD indices. The wall motion filter, followed by the PRF, significantly influences the 3D PD indices, but only when the flow velocities are less than 20 cm per second [86]. An ex vivo experiment showed that PD overestimates the flow-volume data of small vessels, similar to 2D PD, especially when the spatial resolution and Doppler settings are not optimized [87]. A clinical study also showed significant differences in the flow-volume indices when calculated using PD and HDF [88]. Correction factors remain unestablished, because a gold-standard confirmation method that accurately measures the true blood volume in vivo does not exist [61]. Lastly, the acquisition speed and angle are defined. A faster acquisition speed reduces all 3D PD indices, especially the vascularization-flow index [89]. Post-scan adjustment of the Doppler settings should be avoided because doing so may create faulty images. The initial display of the flow-volume data enables the operator to quickly navigate, measure (the ratio, area, and volume), convert the data to nomogram equivalents (i.e., the gestational age), reslice, produce, and save all of the standard image planes required for diagnosis. The manufacturers’ software, such as 4D View or ViewPoint (GE Medical Systems–Kretztechnik, Zipf, Austria), is increasingly being preferred to the conventional digital imaging and communication in medicine because of their ability to integrate flow-volume data and metadata (data of the acquired data) using various algorithmic analyses to obtain the most personalized clinical interpretations with the least image artifacts [90,91].

The four common types of Doppler artifacts mainly arise from tissue gradients and motion. They are (a) twinkle artifacts (occurring behind reflectors such as the fetal skull plate), (b) edge artifacts (occurring along strongly reflective interfaces, like the fetal long bone), (c) flash artifacts (caused by tissue or fluid motion, such as fetal movement, maternal bowel/breathing movement, or slipping of the transducer), and (d) pseudoflow (resulting from the nonvascular flow of other fluids, for example, urinary jets in the maternal bladder or ascites). With the standardized limit of (flow) detection (LOD) threshold, these artifacts are not depicted in confirmatory PWD [92]. Lowering the LOD increases the artifacts, whereas increasing the LOD limits the detection capability. There is a higher tendency for flash artifacts from HDF compared with CD and PD; however, this can be minimized by using optimal Doppler settings [93]. The novel matrix-array transducer can reduce motion artifacts by enabling faster volume rates and semi-interactive acquisition. Standardization of the image display is possible with simultaneous display of two orthogonal morphological planes (biplane mode) [94,95].

### 3.9. Current Challenges

Although prenatal 3D PD may aid in the personalized prediction of preeclampsia and the POC diagnosis of PAS, it should be selectively used to maximize its cost benefits [96]. The technology is highly dependent on the operator; hence, low reproducibility is the most important challenge to incorporating 3D PD in prenatal care. Although proper training can reduce the variability of 3D US volumetric measurements, the training efficacy is still impacted by the task-specific learning curve and the prior experience of the trainees. For example, the learning curve to measure the fetal frontomaxillary facial angle at 11 to 13 weeks of gestation using specially acquired 3D US volume data reaches its competency after a median of 90 training cases, with a broad range of up to 140. The required number of training cases to reach competency was substantially lower (40) for trainees with extensive prior experience of nuchal translucency measurements [97]. Compared with a protocol-based imaging modality (such as magnetic resonance imaging), volumetric measurements obtained with 3D US consistently have greater variability than magnetic resonance imaging measurements, even in experienced hands [98]. Our literature search did not identify a publication directly addressing the learning curve or a validated training protocol for prenatal 3D PD. The lack of training and standardization of the techniques create major differences between publications and between research groups.

Concern about the safety of the developing fetus has always been paramount since the inception of ultrasound technology. During 3D US acquisition, a fetus is exposed to an ultrasound beam for only a few seconds. This exposure is not different from real-time B-mode scanning, and thus prenatal assessment with 3D US should be as safe as a standard B-mode scan. However, Doppler uses a higher intensity power than B-mode ultrasound. Animal studies have shown functional and anatomical bioeffects from prenatal exposure with Doppler [99]. Therefore, diagnostic ultrasound equipment has been regulated by controlling the output pulse and continuous ultrasound waves for all of its applications, including 3D PD. The aim is to keep the thermal index and mechanical index lower than 1.0, which is theoretically safe for prenatal use [100]. Although 3D PD is theoretically safe during pregnancy, it is nevertheless advisable to restrict its use to situations where the possible added benefits outweigh the potential risks. The clinical ramifications of the current challenges of adopting 3D PD to prenatal assessments are detailed in Table 1.

### 3.10. Future Prospects

The International Society of Ultrasound in Obstetrics and Gynecology recently acknowledged the potential of machine-guided acquisition of the standard fetal biometric plane, pattern recognition of anatomical aberrations, and quality assurance of fetal scanning [122,123]. Deep learning automatically analyzes unstandardized prenatal 2D US images and accurately predicts postnatal outcomes [124]. Predictions can be further improved by allowing the machine to access a larger pool of outcome data [125]. It is possible that artificial intelligence will soon be integral to prenatal 3D US [126,127]. Our theoretical conjectures are outlined in Table 2.

## 4. Conclusions

In theory, 3D PD may offer more additional information than the standard prenatal real-time 2D US and Doppler. It may also facilitate teaching and research. This is because the 3D PD technology uniquely archives flow-volume data for offline navigation and analysis with different algorithms, which is not possible with real-time Doppler ultrasound. However, 3D PD has a low reproducibility due to operator dependency, and the technology remains within the areas of research and clinical trials with various levels of experimentation. More time is needed for the maturation of the technology and the establishment of standardization and quality-assurance protocols. As for the current practice, the use of 3D PD as a stand-alone clinical or research tool—even in expert hands—cannot be recommended.

## Figures and Tables

**Table 1:** Current challenges of three-dimensional sonoangiography for prenatal assessment. Abbreviations: 3D-FMBV, 3D-fractional moving blood volume; 3D PD, three-dimensional power Doppler; ALARA, as low as reasonably achievable; FVV, fetal vascular volume; MI, Mechanical Index; PAS, placenta accreta spectrum; PBVV, placental bed vascular volume; PE, preeclampsia; POC, point-of-care; PPV, positive predictive value; NPV, negative predictive value; SPTA, spatial peak temporal average; TI, Thermal Index; TOP, termination of pregnancy.

Challenges	Rationale	Clinical Ramifications
Critical appraisal; case reports.	Feasibility Experienced operators	Low reproducibility Limited clinical translation [101]
Critical appraisal; cross-sectional studies (prevalence of 3D PD findings in study cohorts)	No proof of cause and effect Experienced operators	Moderate reproducibility Limited clinical translation [101]
Critical appraisal; case–control studies (cause–effect comparison from 3D PD cases, and matched controls in study cohorts)	Odds ratio; absolute and relative risks are defined from a small representation of the entire population.	Moderate reproducibilityReasonable clinical translation [101]
Lack of high-quality longitudinal (cohort and panel) studies, randomized controlled trials, meta-analysis, and systematic review	Absence of consensus for the following: Target condition and terminology Performance matrix; sensitivity, specificity, PPV and NPV In vivo gold-standard confirmatory method [89]	Postnatal confirmation; either with standard contrast-imaging techniques or autopsy [53,102,103] In vivo ultrasound contrast agents [104,105,106]
Prediction of PE from first-trimester 3D PD placental assessment	Operator-dependent process; lack of standardization and quality assurance; quality of flow-volume data [61,62,107] Standardized indices, i.e., maternal PBVV (arcuate, radial, basal, and spiral arteries), FVV (umbilical and villous vasculature), and 3D-FMBV [108,109,110]	Powerful equipment [95] Machine automation [111] Validation in different study populations, algorithms, and clinical impacts [63]
Clinical interpretation of flow-volume data	Suboptimal acquisition; faulty results Over-detection; unnecessary anxiety, investigation, and TOP [112] Under-detection; missed diagnosis [113] Software conflicts [114] Metadata; tissue harmonics	Avoid scanning too early or without proper indications [115] Full disclosure of 3D PD for learning and research purposes Cautious interpretation
Best practice and standard recommendations for 3D PD in 11 to 13 +6 weeks of gestation.	Balancing early PE prediction vs. 3D PD safety Lack of evidence; guided by expert consensus [116] Minimal risks; TI is related to exposure to PWD, which is not used in 3D PD; MI is related to output intensity, which is defaulted at < SPTA 240 mW/cm^2^ in most obstetric Doppler settings [117] Large variation of equipment; real-life performance and interactive feedback	Avoid 3D PD during <11 weeks of gestation, especially for learning and research purposes only Strict adherence of ALARA principle
Extrapolation of POC 3D PD for PAS.	Centralization Operator-dependent process [118]	Customized phantom training [119] Algorithmic/scoring approach [72]
Additional costs Resource allocation Research funding	Equipment upgrades, additional training, longer examination time, and workflow ergometrics	Individual health economic analyses [120,121,122]

**Table 2:** Theoretical conjectures of three-dimensional sonoangiography for prenatal assessment. Abbreviations: 3D PD, three-dimensional power Doppler; AI; artificial intelligence; IUGR, intrauterine growth restriction.

Issues	Rationale	Future Prospects
Standardization 3D PD acquisition Prediction of IUGR from second-trimester placental assessment Matrix array transducer; rapid acquisition with Biplane interactive image standardization	Non-uniform flow velocities of the entire placenta [6] Different cardiac outputs at various gestational ages Cardiovascular impacts of fetal conditions Impacts of novel Doppler modality, for example, slow-flow modalities	Machine automation [123,126] Robotic transducer holder and optical sensor [128] Machine instant recognition and assurance of the acquired flow-volume data [124] Automated segmentation of large-volume data to detect anatomical aberrations [129]
Machine-related changes in the following: Training Clinical workflow Medical ethics and liabilities	Complete replacement of human operators by machine [130] Liberal use of AI negatively impacts the skills of human operators [131] Training on handling of the technological novelties, and not patient-centered care; ‘Doctor–Patient–Machine’ relationship [132] Intellectual prioritization over the primary application [133]	Supervised use of AI [133] Human-controlled access to different databases Locked application to prevent automated adaptations

## Data Availability

The data that support the findings of this study are available from the corresponding author upon reasonable request.
